# Split-Thickness Skin Graft Versus Conservative Management for Deep Partial-Thickness Burns: A Systematic Review and Meta-Analysis

**DOI:** 10.7759/cureus.117230

**Published:** 2026-09-30

**Authors:** Rahul Dubepuria, Deepak Krishna, Varsha R Choudhary, Suresh K Thanneeru, Amit Agrawal

**Affiliations:** 1 Department of Burns and Plastic Surgery, All India Institute of Medical Sciences, Bhopal, Bhopal, IND; 2 Department of Obstetrics and Gynaecology, Gandhi Medical College, Bhopal, IND; 3 Department of Pediatric Surgery, All India Institute of Medical Sciences, Bhopal, Bhopal, IND; 4 Department of Neurosurgery, All India Institute of Medical Sciences, Bhopal, Bhopal, IND

**Keywords:** deep partial thickness burns, second degree deep burns, split thickness skin grafting, wound care management, wound dressings

## Abstract

Superficial burns heal spontaneously, whereas deep burns require grafting. Deep partial thickness (DPT) burns can heal spontaneously but may take longer and can form hypertrophic scars and contractures. The choice between split-thickness skin grafting (STSG) and conservative management for DPT burns remains a dilemma. This systematic review and meta-analysis compares the efficacy and safety of both procedures. PubMed, Cochrane Central Register of Controlled Trials (CENTRAL), Scopus, and ScienceDirect databases were systematically searched for relevant articles. Randomized controlled trials (RCTs), quasi-experimental studies, and cohort studies were included. Case reports, case series, editorials, animal studies, and non-English literature were excluded. The meta-analysis was performed using a random-effects model in RevMan 5.4. Seven studies, including 650 patients, were included in the final analysis. The pooled analysis showed that the STSG group had significantly reduced wound-healing time (standardized mean difference (SMD): -2.01 (-3.80 to -0.22), p=0.03) and a shorter hospital stay (SMD: -3.75 (-6.98 to -0.52), p=0.02). No significant differences were found in wound infection rates, long-term scarring, or the need for reconstructive procedures at a later date. STSG offers a shorter wound-healing time and a reduced hospital stay compared with conservative management for DPT burns. However, due to high heterogeneity and a limited number of studies, further RCTs will help provide better evidence.

## Introduction and background

Burns are representative of a global health crisis, with over 90% of burns occurring in lower-middle-income countries, where resources are limited [1-3]. According to the WHO, there are an estimated 180,000 burn deaths per year, most of which occur in low- and middle-income countries [4]. Burns are classified into superficial (involving epidermis), superficial partial-thickness burns (involving epidermis and papillary layer of dermis), deep partial-thickness burns (involving reticular layer of dermis), and full-thickness burns (involving epidermis and complete dermis). Deep partial-thickness (DPT) burns represent a clinical "grey zone" in burn care. Unlike superficial burns that heal spontaneously or full-thickness burns that require grafting, DPT burns can heal through epithelialization from skin appendages but often take longer than three weeks. The development of successful topical antibacterials for the care of burn wounds in the mid-1960s made another alternative possible [5]. Prolonged healing time is strongly associated with hypertrophic scarring, contractures, and functional disability.

While early excision and split-thickness skin grafting (STSG) are often claimed to have shortened hospital stays and reduce scarring, surgery introduces donor-site morbidity and increased immediate costs. Conversely, conservative management with advanced dressings avoids surgery but may lead to prolonged inflammation. The choice of STSG versus conservative management may depend on multiple factors such as burn depth, total body surface area involved, and anatomical sites like the hands and face. Currently, there is no definitive consensus on the optimal threshold for surgical intervention in DPT burns due to the limited number of studies and variability in burn depth, area, and anatomical location; most surgeons rely on individual case-by-case evaluation. This systematic review aims to synthesize high-quality evidence to compare these two fundamental approaches.

## Review

Methods

This systematic review was conducted in accordance with the Preferred Reporting Items for Systematic Reviews and Meta-Analyses (PRISMA) guidelines [6]. The review was registered in PROSPERO (CRD42025631420), and ethical approval from the institute was not required, as this is a secondary synthesis of the published literature.

Eligibility Criteria

Studies were selected using the Participants, Intervention, Comparison, and Outcomes (PICO) framework to evaluate the clinical efficacy and safety of STSG compared with conservative management with dressings in pediatric and adult patients with DPT burns. 

Participants (P): Patients of any age (children and adults) diagnosed with deep partial-thickness burns (confirmed clinically or via laser Doppler imaging); Intervention (I): Surgical management involving early or delayed excision followed by split-thickness skin grafting (STSG); Comparator (C): Conservative management using dressings (e.g., silver-based, paraffin gauze, or biosynthetic dressings) without skin grafting; Outcomes (O): (a) Primary: Healing time, infection rate (wound infection or sepsis), and length of hospital stay (LOS); (b) Secondary: Long-term scarring (Vancouver Scar Scale or Patient and Observer Scar Assessment Scale scores), range of motion/disability, and cost-effectiveness.

Study Design

The review included randomized controlled trials (RCTs), prospective cohort studies, and retrospective comparative studies.

Information Sources

A systematic literature search was conducted across PubMed, the Cochrane Central Register of Controlled Trials (CENTRAL) Library, Scopus, and ScienceDirect databases to identify relevant studies from inception till 8th of April 2026.

Search Strategy

The search terms listed in Table 1 were utilized across the above-mentioned databases. In addition, the reference lists of the included articles were reviewed for relevant articles (citation searching) and added as "other" sources.

**Table 1:** Search strategy

Database	Search strategy
PubMed	(("partial thickness burn*"[All Fields] OR "second degree burn*"[All Fields]) AND ("early excision"[All Fields] OR "skin grafting"[All Fields]))
ScienceDirect	Title, abstract, keywords: ("partial thickness burn" OR "second degree burn") AND ("early excision" OR "skin grafting")
SCOPUS	TITLE-ABS-KEY ( "partial thickness burn*" OR "second degree burn*" ) AND TITLE-ABS-KEY ( "early excision" OR "skin grafting" OR "split thickness skin graft*" )
COCHRANE	burn* AND ("early excision" OR "skin grafting") in Title Abstract Keyword

Study Selection

Duplicated records were identified electronically using EndNote software (Clarivate, London). Three investigators (SKT, RA, and AA) independently screened the titles and abstracts based on the inclusion and exclusion criteria mentioned below, and relevant articles were selected for full-text screening. Conflicts were resolved through consensus.

Inclusion Criteria

Studies comparing STSG with conservative management without skin grafting in DPT burns were included (irrespective of the burn etiology and total body surface area of burns). Randomized controlled trials (RCTs), prospective cohort studies, retrospective comparative studies, and articles in English literature were included.

Exclusion Criteria

Case reports, case series, reviews, animal studies, and studies involving full-thickness grafts or skin substitutes as a definitive replacement for grafting were excluded.

Data Extraction

Five investigators (RA, SKT, DK, VRC, and AA) independently assessed and sorted the full-text articles. The excluded articles were reported with reasons. From the included articles, the data were extracted from a predesigned proforma. The details of the data extracted were study characteristics (author, year, country, study design), participant data (sample size, age (mean/range), total body surface area (TBSA) (%), burn etiology, anatomical location), intervention details (timing of surgery, graft thickness, donor site care), control details (type of dressings used, frequency of changes), outcome data (mean/standard deviation (SD) for continuous variables (healing time, length of hospital stay (LOS), scar scores); events/totals for dichotomous variables (infection rates)). The authors were contacted for missing data, and any discrepancies were resolved by consensus.

Outcomes

The primary outcomes evaluated were healing time, infection rate (wound infection or sepsis), and length of hospital stay (LOS). The secondary outcomes evaluated were long-term scarring (Vancouver Scar Scale or Patient and Observer Scar Assessment Scale scores), range of motion/disability, and cost-effectiveness.

Risk of Bias and Quality Assessment

The methodological quality of the included studies was assessed using the Joanna Briggs Institute (JBI) Critical Appraisal Tools according to the study design [7]. Five authors (RA, SKT, DK, VRC, and AA) independently assessed the methodological quality of the studies, and any disagreements were resolved through consensus. Each study was categorized as High, Moderate, or Low quality based on the percentage of "Yes" responses (e.g., >70% = High).

Effect Measures

Continuous outcomes, such as healing time and length of stay, were analyzed using mean difference (MD) or standardized mean difference (SMD) with 95% confidence intervals (CI). For dichotomous outcomes, such as infection rates, risk ratio (RR) was calculated with 95% CI.

Synthesis Methods

The meta-analysis was conducted using Review Manager (RevMan, version 5.4: The Cochrane Collaboration) [8]. Heterogeneity was assessed using the I^2^ statistic, and values greater than 70% were considered indicative of high heterogeneity. Publication bias was not assessed because the number of articles was fewer than 10.

Results

Study Selection

According to the search strategy outlined in Table 1, we retrieved 460 records. Three articles were added after a citation search of the included articles. After removing 124 duplicates, the remaining 336 articles were considered for title and abstract screening. After screening, 11 articles were selected for full-text screening. Seven articles were excluded; the reasons are provided in Table 2.

**Table 2:** Studies excluded with reasons

Author	Reason for exclusion
Ayaz et al., 2019 [9]	Early vs delayed grafting; no comparison with non-operative management
Batra et al., 2021 [10]	No comparative arm of grafting
Chu et al., 1991 [11]	Animal study
Coruh and Yontar, 2012 [12]	No conservative arm
Frist et al., 1985 [13]	Excluded as there is no comparison
Li et al., 2018 [14]	No grafting arm
Sun et al., 2024 [15]	Xenomic grafts were used

The remaining four articles and three articles from citation search were selected for qualitative synthesis. One included article [5] lacked sufficient material for meta-analysis; hence, six articles were included for meta-analysis [16-21]. The study selection process is illustrated in Figure 1 using the Preferred Reporting Items for Systematic Reviews and Meta-Analyses (PRISMA) flow chart. 

**Figure 1:**
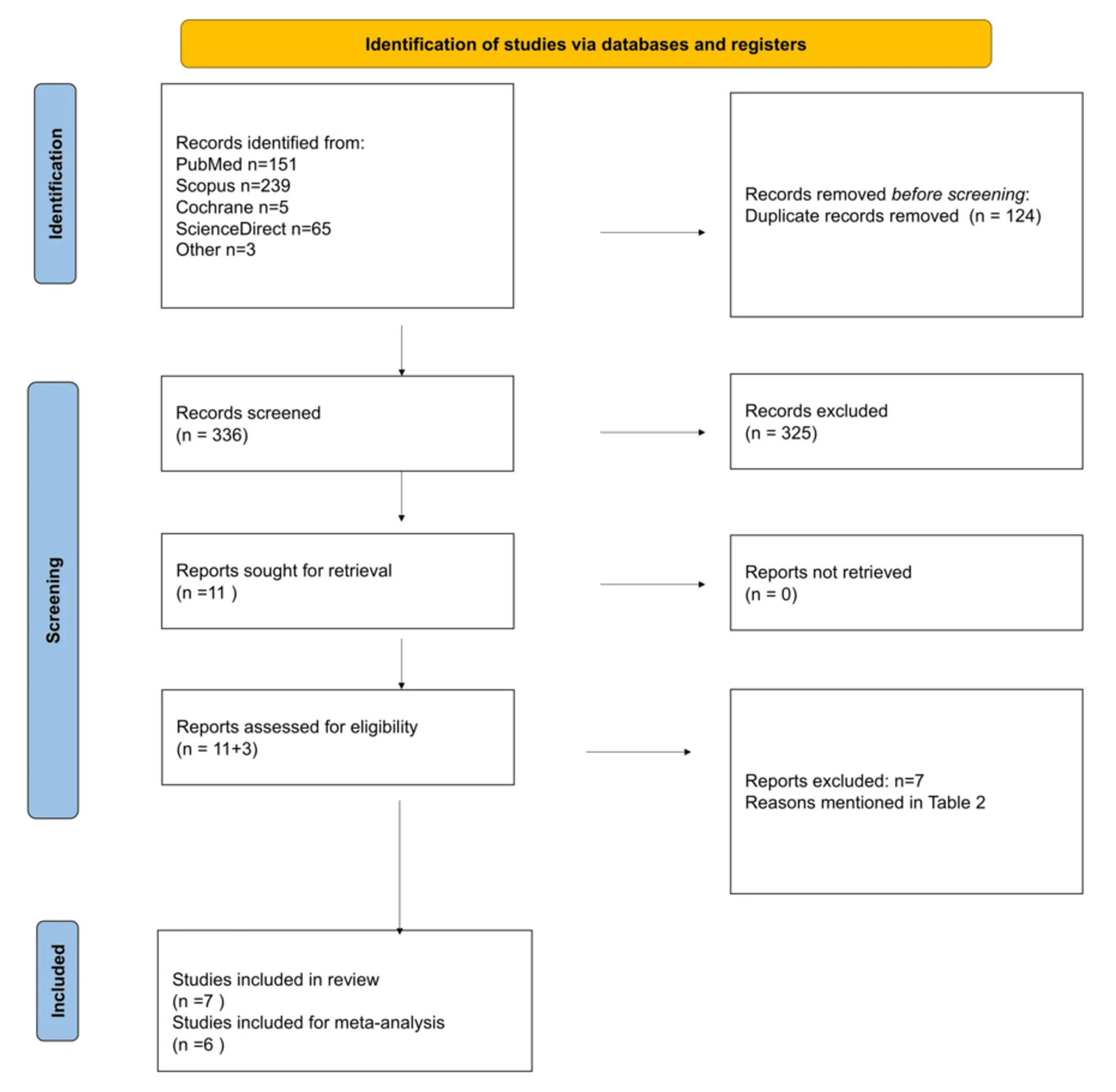
PRISMA flow diagram PRISMA: Preferred Reporting Items for Systematic Reviews and Meta-Analyses.

Study Characteristics

Among the seven included studies, three are from the United States, and one each from Poland, Lithuania, Pakistan, and Iran. Three studies are randomized controlled trials (RCTs), two are quasi-experimental studies, and two are retrospective studies. One study treated only pediatric burns, one covered both adults and pediatric patients, and the remaining studies focused on adults. Across all articles, the total number of subjects was 650, of which 280 received excision and grafting, and the remaining 370 were managed conservatively. Three studies used antiseptic dressings for conservative management, and the remaining three used antimicrobial dressings for conservative management. Three studies focused exclusively on hand burns, and one study focused exclusively on the buttocks and posterior trunk. The details of the study characteristics are summarized in Table 3.

**Table 3:** Characteristics of the included studies NA: Not applicable; RCT: randomized controlled trial; TBSA: total burn surface area; BSA: burn surface area.

Study ID	Country	Study design	Study population	Experiment vs control	Inclusion/exclusion- methods	Results	Remarks
Bruke et al., 1976 [16]	USA	Quasi-experimental study	Children and adults (152 patients with 226 burned hands)	76 patients (30 children and 46 adults) with deep second- and third-degree burns who underwent excision and grafting; 76 patients (30+46) who were treated with silver nitrate alone without excision	Deep second- and third-degree burns over the hand. Superficial burns (healing within 3 weeks) and amputations were excluded	Primary excision significantly reduced the need for reconstructive surgery (35% in grafting vs 97% in controls), and there are improved functional and cosmetic results	Full-thickness burns were excluded in our review
Edstrom et al., 1979 [5]	USA	RCT	Adults - hand injuries (222)	Excision and grafting vs conservative management with scarlet red dressing	Full-thickness and superficial burns were excluded. Burns over hands. The first 10 days were managed with topical silver sulphadiazine.	96 unhealed hands at 10 days. Reported that there is no significant difference between the two groups; however, no data have been provided.	Insufficient data for meta-analysis
Engrav et al., 1983 [17]	USA	RCT	47 adults <20% BSA deep partial-thickness burns	Early excision and grafting (22) vs non-operative treatment with silver sulfadiazine (25)	Flames/scalds involving <20% TBSA and healthy adults; those associated with trauma, full-thickness, and superficial thickness burns were excluded	Early excision and grafting group had shorter hospital stay (16.4 vs 25 days) and less hypertrophic scarring	NA
Kaźmierski et al., 2007 [18]	Poland	Retrospective cohort study	Children – injuries excluding face, armpits, palms, etc.	Excision and grafting vs four non-operative strategies including dermabrasion, hydrocolloid/enzymatic/hydrofiber dressing	Deep partial thickness burns with 3-40% BSA. Excluded were special sites like face/palms, etc and patients whose wounds are not healing by 2 weeks of conservative management (converted to grafting)	Excision and grafting is recommended for more than 20% BSA burns, and conservative management has similar results with grafting if the burn area is less than 20%	NA
Maslauskas et al., 2009 [19]	Lithuania	RCT	Patients with deep partial thickness burns over hands	Partial thickness skin grafting (n=24) vs conservative treatment with silver sulfadiazine(n=25)	Deep partial-thickness burns over hands <40% TBSA	Grafting resulted in fewer infections (27.5% vs 84.6%), better Vancouver scores (3.65% vs 6.77%), and less functional deficiency at 12 months (12% vs 23%)	NA
Saeed et al., 2024 [20]	Pakistan	Retrospective cohort study	Adult thermal burns with less than 30% TBSA with deep partial thickness burns	Early excision and grafting vs non-operative management with antimicrobial dressing	Thermal burns with less than 30% TBSA deep partial thickness burns were included; those presented more than 24-h post injury, associated trauma, and contaminated wounds were excluded	Early grafting has superior outcomes in faster healing, shorter hospital stays, and fewer hypertrophic scars	NA
Zanganeh et al., 2022 [21]	Iran	Quasi-experimental study (even though the authors mentioned the study as a cross-sectional analytical study)	Patients with burns over the posterior trunk and buttocks with deep second-degree burns	Grafting vs non-operative management (silver sulfadiazine or mupirocin)	Deep second-degree posterior trunk and buttocks burns were included; excluded were deep burns and chemical or electrical burns, and patients with diabetes aged more than 70 years	Mean scar (Vancouver scores) was higher in the skin grafting group compared to the non-graft group.	NA

Main outcomes

Wound Healing Time

Two studies, Kaźmierski et al. [18] and Saeed et al. [20], reported healing time. A total of 231 patients were tested: 78 in the STSG group and 153 in the conservative group. SMD was used to address the methodological and temporal variations across the studies. The pooled meta-analysis using a fixed-effect model (a fixed-effect model was chosen instead of a random-effects model, even with high heterogeneity, to avoid the mathematical instability and unreliability associated with estimating between the study variance using small study subsets), a statistically significant difference was observed with the STSG group having less healing time (SMD=-1.85; 95% CI of -2.20 to -1.50; p<0.00001) with I2 of 96% (Figure 2).

**Figure 2:**
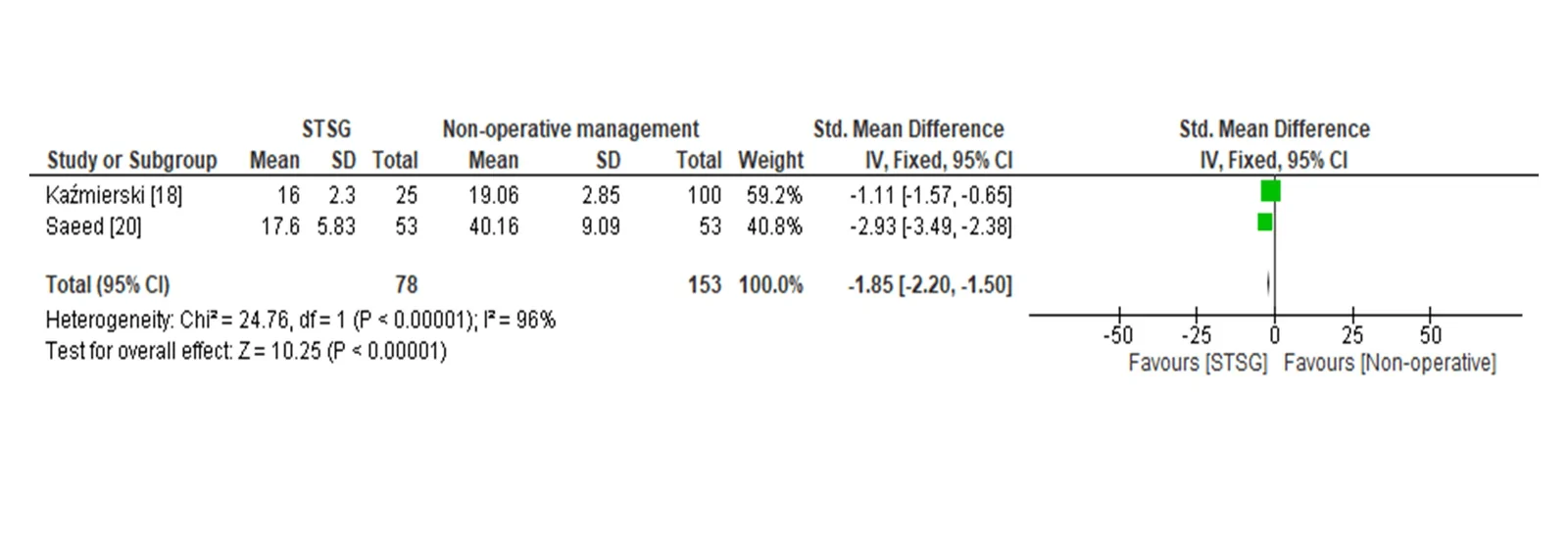
Forest plot of wound healing time: split-thickness skin grafting (STSG) vs conservative management STSG: Split-thickness skin grafting.

Wound Infection Rate

Three studies, Maslauskas et al. [19], Kaźmierski et al. [18], and Saeed et al. [20], reported wound infection rates. A total of 310 patients were studied, of whom 118 underwent STSG, and the remaining 192 were managed conservatively. The overall wound infection rate did not differ significantly between the STSG and conservative groups, RR of 0.95; 95% CI of 0.22 to 4.07; p=0.94. However, the subgroup analysis of the RCTs demonstrated a significantly lower wound infection rate in the STSG group (RR of 0.33; 95% CI of 0.19 to 0.55; p<0.0001), whereas the non-RCTs demonstrated no significant difference(RR of 2.02; 95% CI of 0.91 to 4.47; p=0.08, I2 =0%). Heterogeneity was 86% across all studies combined, but there was no significant heterogeneity in non-RCTs (Figure 2).

**Figure 3:**
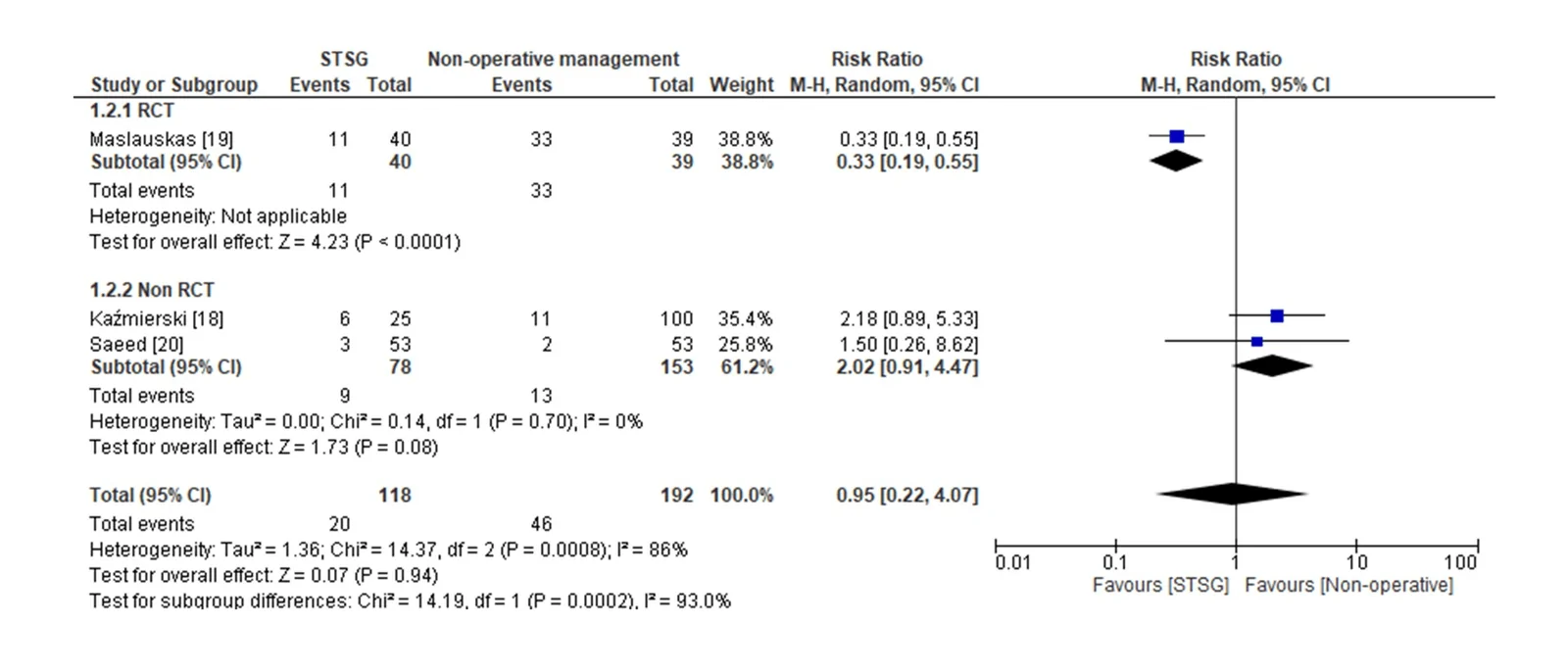
Forest plot of wound infection rate: split-thickness skin grafting (STSG) vs conservative management

Length of Hospital Stay

Two studies, Engrav et al. [17], an RCT, and Saeed et al. [20], a non-RCT, reported results on length of hospital stay between the groups. The pooled analysis showed a significant reduction in length of hospital stay in the STSG group compared with the conservative group, with an SMD of -2.57 (95% CI: -3.02 to -2.11) and p<0.00001. However, there was significantly high heterogeneity (I^2^=95%), p<0.00001 (Figure 3).

**Figure 4:**
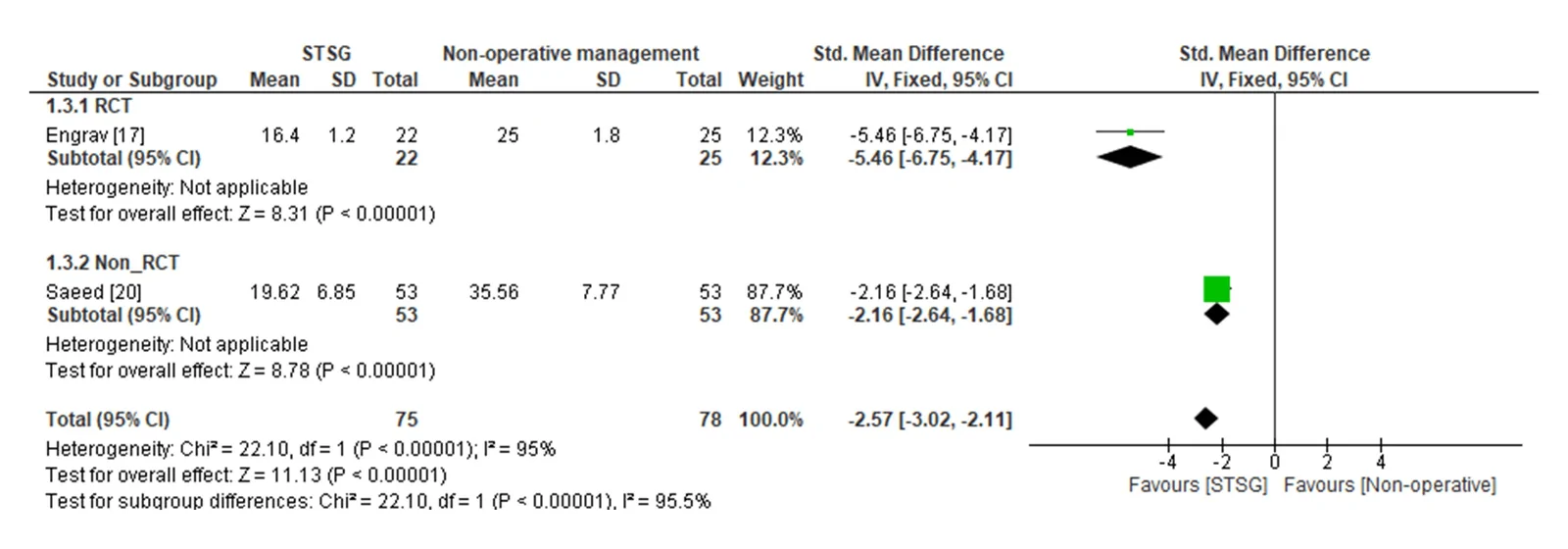
Forest plot of length of hospital stay: split-thickness skin grafting (STSG) vs conservative management

Secondary outcomes

Long-Term Scarring

Three studies - Maslauskas et al. [19], an RCT, Kaźmierski et al. [18] and Zanganeh et al. [21], both of which are non-RCTs - reported on long-term scarring results based on the Vancouver Scar Scale (VSS). There was no significant difference between the groups in long-term scarring, with an SMD of 1.30 (95% CI=-1.33 to 3.64) and p=0.10. There was moderate heterogeneity between the studies, with I^2^ of 62.2%. In the subgroup analysis, the RCT by Maslauskas et al. [19] demonstrated a significant difference in scarring rates, with STSG having less scarring. In non-RCTs, Zanganeh et al. [21] showed less scarring in the conservative group, resulting in heterogeneity in the results (Figure 4).

**Figure 5:**
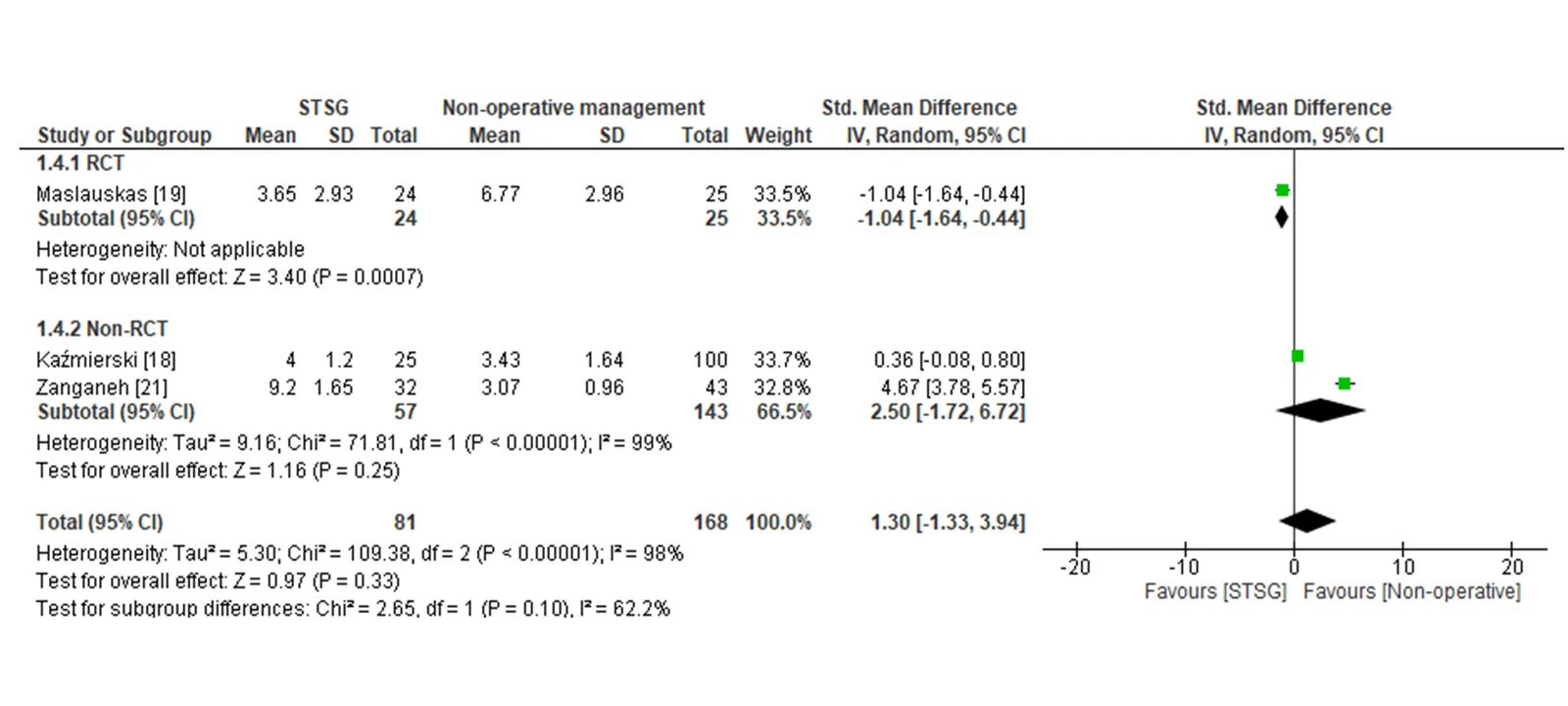
Forest plot long-term scarring: split-thickness skin grafting (STSG) vs conservative management

Requirement of Reconstructive Procedures for Disability

Two studies, Bruke et al. [16], a quasi-experimental study, and Engrav et al. [17], an RCT, reported the need for reconstructive procedures at follow-up. The pooled results showed no significant difference between the groups, with an RR of 0.26 (0.03 to 2.03), p=0.20. There was moderate heterogeneity (I²=53%) (Figure 5).

**Figure 6:**
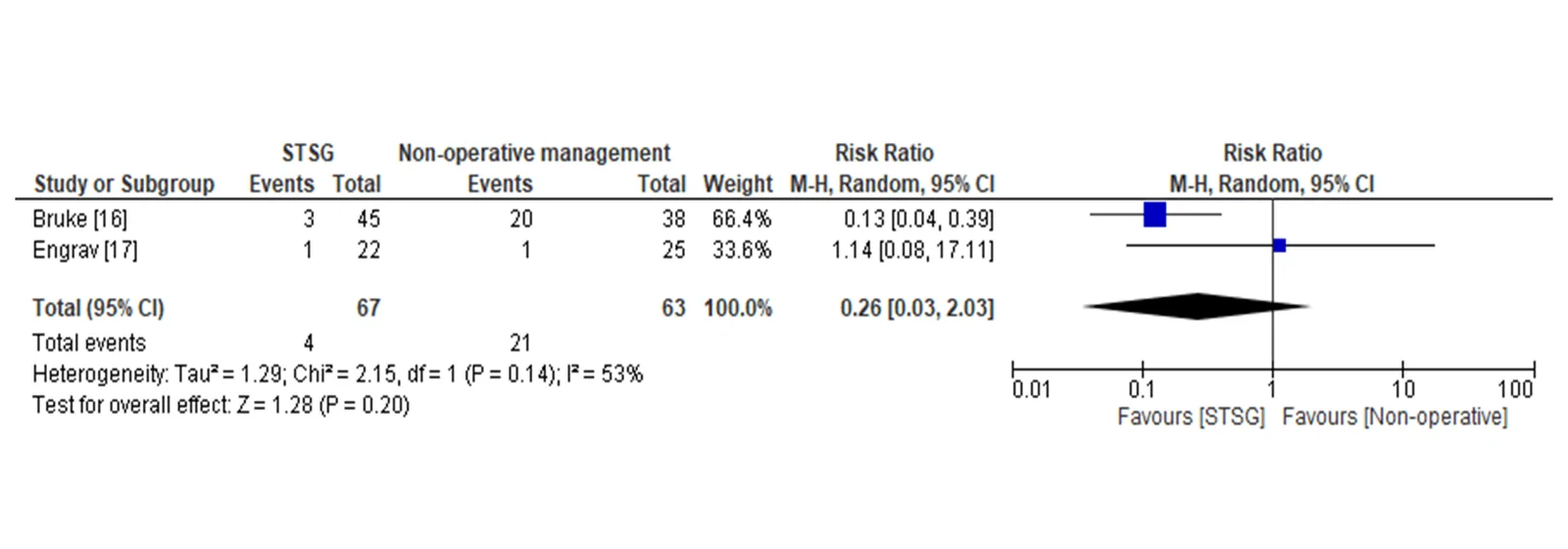
Forest plot of requirement for reconstructive procedures due to disability: split-thickness skin grafting (STSG) vs conservative management

Cost Effectiveness

Only one study, Engrav et al. [17], reported cost-effectiveness, with the STSG group having significantly lower expenditure than the conservative group (p=0.05). As only one study provided data, a meta-analysis was not conducted.

Risk of bias

The methodological quality of the seven included studies was assessed using the JBI critical appraisal tool, according to study type [22,23]. Overall, quality ranged from moderate to high. The limitations included blinding of participants (which may not be possible, as patients will know if grafting is being performed), blinding of treatment delivery, and blinding of outcome assessors. The retrospective cohort and quasi-experimental studies were of high quality. The summarised risk of bias for RCTs is mentioned in Table 4.

**Table 4:** Joanna Briggs Institute (JBI) critical appraisal for randomised controlled trials (RCTs) [23] Questions: Q1: Randomisation; Q2: Concealment; Q3: Baseline Similarity; Q4: Participant Blinding; Q5: Deliverer Blinding; Q6: Assessor Blinding; Q7: Identical treatment; Q8: Follow-up; Q9: ITT analysis; Q10: Measuring same way; Q11: Measurement reliability; Q12: Stats; Q13: Design.

Author	Q1	Q2	Q3	Q4	Q5	Q6	Q7	Q8	Q9	Q10	Q11	Q12	Q13
Edstrom et al. [5]	Yes	Unclear	Unclear	No	No	Unclear	Yes	Yes	Yes	Yes	Yes	Yes	Yes
Engrav et al. 1983 [17]	Yes	Unclear	Yes	No	No	Unclear	Yes	No	Yes	Yes	Yes	Yes	Yes
Maslauskas et al. [19]	Yes	Unclear	Yes	No	No	Unclear	Yes	Yes	Yes	Yes	Yes	Yes	Yes

The summarised risk of bias for cohort studies is mentioned in Table 5.

**Table 5:** Joanna Briggs Institute (JBI) critical appraisal for cohort studies Questions: Q1: Group similarity; Q2: Measurement similarity; Q3: Validity of measurement; Q4: Confounding identified; Q5: Confounding strategies; Q6: Outcome-free start; Q7: Outcome validity; Q8: Follow-up time; Q9: Follow-up complete; Q10: Loss to follow-up strategy; Q11: Stats.

Author	Q1	Q2	Q3	Q4	Q5	Q6	Q7	Q8	Q9	Q10	Q11	
Saeed et al. [20]	Yes	Yes	Yes	Yes	Yes	N/A	Yes	Yes	Yes	Unc	Yes	
Kaźmierski et al. [18]	Yes	Yes	Yes	No	No	N/A	Yes	Yes	Yes	No	Yes	

The summarised risk of bias for quasi-experimental studies is mentioned in Table 6.

**Table 6:** Joanna Briggs Institute (JBI) critical appraisal tool for quasi-experimental study design [22] Questions: Q1. Cause and Effect orientation; Q2: Group similarity; Q3: Treatment similarity; Q4: Control group; Q5: Measurement intervals; Q6: Follow up completeness; Q7: Measurement similarity; Q8: Validity of measurement; Q9: Statistics

Author	Q1	Q2	Q3	Q4	Q5	Q6	Q7	Q8	Q9	
Bruke, 1976 [16]	Yes	Yes	Yes	Yes	No	Yes	Yes	Yes	No	
Zanganeh, 2022 [21]	Yes	No	Yes	Yes	No	Yes	Yes	Yes	Yes	

Discussion

Principal Findings and Clinical Implications

This systematic review and meta-analysis provides insight into the dilemma of STSG vs conservative management for DPT burns. Overall, the aggregated data showed an advantage of STSG over conservative management. Even though there is a significant difference in healing time in STSG compared to conservative management from the pooled analysis, Kaźmierski et al. [18] reported that the results are significant only in patients with more than 20% BSA. Kaźmierski et al. [18] reported a mean healing time of 19.06 days, whereas Saeed et al. [20] reported a mean healing time of 40.16 days, creating more heterogeneity.

Our meta-analysis showed no significant overall difference in wound infection rates between the groups. However, the RCT by Maslauskas et al. [19] showed a significant difference between the STSG and conservative groups. In non-RCTs, there might be selection bias, with smaller, cleaner wounds selected for the conservative group.

The length of hospital stay serves as a surrogate marker for better treatment efficacy and is directly related to the socioeconomic burden of burn injuries, especially in developing countries. Our meta-analysis showed a significantly shorter hospital stay in the STSG group. The heterogeneity of the studies can be explained by the difference in study periods of Engrav et al. [17] and Saeed et al. [20], which span four decades. Even though they both showed a reduction in hospital stay, Engrav et al. [17] had a longer hospital stay in the conservative group, probably due to limited burn infrastructure and hospital protocols in the 1980s, which have significantly evolved by 2024. These results should be interpreted with caution, as this might change with institutional protocols, and the majority of patients may be managed on an outpatient department (OPD) basis.

Long-term scarring in burns contributes to the chronic morbidity of burns and reduced quality of life in burn survivors. Our meta-analysis did not find any significant difference between the groups; however, this should be interpreted with caution. The RCT by Maslauskas et al. [19] showed a significant reduction in scarring in the STSG group. The non-RCTs by Kaźmierski et al. [18] did not show any significant difference (might be due to selection bias), but the study by Zanganeh et al. [21] showed significantly less scarring in the conservative group. This study focused exclusively on burns over the posterior trunk and buttocks. The authors noted that this is a special group because the constant pressure in this area prevents scarring or thickening, thereby acting as a tourniquet during follow-ups.

Our meta-analysis did not find any significant difference between the groups in terms of the need for reconstructive procedures during follow-up. However, Bruke et al. [16] reported a significant benefit in the STSG group compared to Engrav et al. [17], where no significant difference was observed. This can be explained similarly to the hospital length of stay, where newer studies had better protocols for burn management, including early mobilization and aggressive physiotherapy, compared to older studies.

Comparison with current literature

Our findings align with the general observations that surgical excision and grafting may accelerate wound healing in DPT burns. However, none of the literature mentioned the donor site morbidity and blood loss associated with surgical procedures. 

Strengths and limitations

The strengths of this review include strict inclusion criteria for deep partial-thickness burns, where literature on this grey area is scarce. Additionally, subgrouping RCTs and non-RCTs to address heterogeneity. The limitations include a small number of eligible articles with small sample sizes, high heterogeneity among the included studies, no clear operational definitions of outcomes such as wound healing time and length of hospital stay, and a moderate risk of bias in RCTs. We could not perform age-specific subgroup analysis on paediatric vs. adult due to limited segregated data among the included studies. While our search strategy included standard terminology such as deep partial thickness burns and split skin thickness grafting, variation in indexing terms like intermediate depth burn or tangential excision may have resulted in additional studies. Another important limitation is the variability in publication eras of the included studies, ranging from the 1970s, when there was a significant change in protocols and infrastructure. 

Future research and explanations for heterogeneity

High heterogeneity is mainly attributed to clinical and methodological variations, including burn size, anatomical location, criteria for hospital stay, and definitions of wound healing. Differences in the publication periods, as mentioned in the limitations section, reflect a major shift in protocol and infrastructure. Further research on the same topic with a larger number of RCTs, focused on particular anatomical regions, stratifying by different body surface area of burns and taking donor area and blood loss into consideration can help in further resolving the dilemma.

## Conclusions

Split-thickness skin grafting offers a shorter healing time and a reduced hospital stay compared with conservative management with dressings for deep partial-thickness burns. There is no significant difference in wound infection rates, long-term scarring, or the need for reconstructive procedures at a later date. However, given the high heterogeneity and high impact on localized anatomical factors, further RCTs and body region-specific studies are needed to compare these approaches with precision.

## References

[1] Peck M, Pressman MA (2013). The correlation between burn mortality rates from fire and flame and economic status of countries. Burns.

[2] Jacobs C, Vacek J, Many B, Bouchard M, Abdullah F (2021). An analysis of factors associated with burn injury outcomes in low- and middle-income countries. J Surg Res.

[3] Goswami P, Sahu S, Singodia P, Kumar M, Tudu T, Kumar A, Sinha PK (2019). Early excision and grafting in burns: an experience in a tertiary care industrial hospital of eastern India. Indian J Plast Surg.

[4] (2023). World Health Organization. Burns. https://www.who.int/news-room/fact-sheets/detail/burns.

[5] Edstrom LE, Robson MC, Macchiaverna JR, Scala AD (1979). Prospective randomized treatments for burned hands: nonoperative vs. operative. Preliminary report. Scand J Plast Reconstr Surg.

[6] Page MJ, McKenzie JE, Bossuyt PM (2021). The PRISMA 2020 statement: an updated guideline for reporting systematic reviews. BMJ.

[7] Hilton M (2024). JBI Critical appraisal checklist for systematic reviews and research syntheses. J Can Health Libr Assoc.

[8] (2020). Review Manager (RevMan). Version 5.4. The Cochrane Collaboration. http://revman.cochrane.org.

[9] Ayaz M, Karami MY, Deilami I, Moradzadeh Z (2019). Effects of early versus delayed excision and grafting on restoring the functionality of deep burn-injured hands: a double-blind, randomized parallel clinical trial. J Burn Care Res.

[10] Batra N, Zheng Y, Alberto EC, Ahmed OZ, Cheng M, Shupp JW, Burd RS (2021). Pediatric treadmill friction burns to the hand: outcomes of an initial nonoperative approach. J Burn Care Res.

[11] Chu CS, McManus AT, Okerberg CV, Mason AD Jr, Pruitt BA Jr (1991). Weak direct current accelerates split-thickness graft healing on tangentially excised second-degree burns. J Burn Care Rehabil.

[12] Coruh A, Yontar Y (2012). Application of split-thickness dermal grafts in deep partial- and full-thickness burns: a new source of auto-skin grafting. J Burn Care Res.

[13] Frist W, Ackroyd F, Burke J, Bondoc C (1985). Long-term functional results of selective treatment of hand burns. Am J Surg.

[14] Li F, Chi YF, Hu Q (2018). Effects of minimally invasive tangential excision in treating deep partial-thickness burn wounds on trunk and limbs in pediatric patients in the early stage post burn (in Chinese). Zhonghua Shao Shang Za Zhi.

[15] Sun J, Su S, Jiao S, Li G, Zhang Z, Lin W, Zhang S (2024). A prospective analysis of the efficacy of phase II autologous skin grafting on deep second‑degree burns on the dorsum of the hand. Exp Ther Med.

[16] Burke JF, Bondoc CC, Quinby WC Jr, Remensnyder JP (1976). Primary surgical management of the deeply burned hand. J Trauma.

[17] Engrav LH, Heimbach DM, Reus JL, Harnar TJ, Marvin JA (1983). Early excision and grafting vs. nonoperative treatment of burns of indeterminant depth: a randomized prospective study. J Trauma.

[18] Kaźmierski M, Mańkowski P, Jankowski A, Harasymczuk J (2007). Comparison of the results of operative and conservative treatment of deep dermal partial-thickness scalds in children. Eur J Pediatr Surg.

[19] Maslauskas K, Rimdeika R, Rapoliene J, Saladzinskas Z, Samsanavicius D, Kaikaris V (2009). The comparison of two methods of treatment evaluating complications and deficiency of functions of hands after deep partial skin thickness hand burns. Medicina (Kaunas).

[20] Saeed S, Sohail M, Bashir MM, Bajwa MS, Nazir U, Khadam M (2024). Comparison of outcome of early tangential excision with autografting versus interactive antimicrobial dressing in deep-partial thickness burn patients: a retrospective analysis. Burns.

[21] Zanganeh M, Keshavarzi A, Dahmardehei M, Ghadimi T, Abdalkhani A, Dehghani A (2022). Skin grafting compared with conservative treatment in patients with deep second-degree burn wounds of the trunk and buttocks. World J Plast Surg.

[22] Barker TH, Habibi N, Aromataris E (2024). The revised JBI critical appraisal tool for the assessment of risk of bias for quasi-experimental studies. JBI Evid Synth.

[23] Barker TH, Stone JC, Sears K (2023). The revised JBI critical appraisal tool for the assessment of risk of bias for randomized controlled trials. JBI Evid Synth.

